# Incidence of viral respiratory infections in a prospective cohort of outpatient and hospitalized children aged ≤5 years and its associated cost in Buenos Aires, Argentina

**DOI:** 10.1186/s12879-015-1213-4

**Published:** 2015-10-24

**Authors:** Débora Natalia Marcone, Lizette O. Durand, Eduardo Azziz-Baumgartner, Santiago Vidaurreta, Jorge Ekstrom, Guadalupe Carballal, Marcela Echavarria

**Affiliations:** Virology Unit and Clinical Virology Laboratory, Centro de Educación Médica e Investigaciones Clínicas “CEMIC” and CONICET, Galván 4102, 1431 FWO Buenos Aires, Argentina; Centers for Disease Control and Prevention, 1600 Clifton Rd, NE, MS A32, Atlanta, GA 30329-4018 Georgia USA; Department of Pediatrics, Centro de Educación Médica e Investigaciones Clínicas “CEMIC”, Buenos Aires, Argentina

**Keywords:** Incidence, Children, Rhinovirus, Respiratory syncytial virus, Influenza, Hospitalized and outpatients, Charges

## Abstract

**Background:**

Although information about the incidence of viral respiratory illnesses and their associated cost can help health officials explore the value of interventions, data are limited from middle-income countries.

**Methods:**

During 2008–2010, we conducted a prospective cohort study and followed ~1,800 Argentinian children aged ≤5 years to identify those children who were hospitalized or who sought care at an emergency room with any acute respiratory infection sign or symptom (e.g., rhinorrhea, cough, wheezing, tachypnea, retractions, or cyanosis). Respiratory samples were obtained for respiratory syncytial virus, influenza, parainfluenza, adenovirus, and metapneumovirus testing by immunofluorescence and for rhinovirus by real-time reverse transcription polymerase chain reaction.

**Results:**

The incidence of respiratory syncytial virus (24/1000 children-years), human metapneumovirus (8/1000 children-years), and influenza (8/1000 children-years) illnesses was highest among hospitalized children aged <6 months and decreased among older children. In contrast, the incidence of rhinovirus was highest (12/1000 children-years) among those aged 6–23 months. In the emergency room, the incidence of rhinovirus was 459; respiratory syncytial virus 352; influenza 185; parainfluenza 177; metapneumovirus 130; and adenovirus 73/1,000 children-years. The total cost of hospitalization was a median of US$529 (Interquartile range, US$362–789).

**Conclusions:**

Our findings indicate that respiratory viruses, in particular rhinovirus, respiratory syncytial virus, metapneumovirus, and influenza may be associated with severe illness causing substantial economic burden.

## Background

Respiratory viruses are the major cause of acute respiratory infections (ARI) which can range from mild and self-limited to severe and even fatal disease. The most frequent viruses related to these infections are respiratory syncytial virus (RSV), influenza, adenovirus, parainfluenza, and metapneumovirus (HMPV) [1–4]. In addition, human rhinoviruses, which have been traditionally described as the cause of the common cold, are also associated with respiratory infections of the lower tract [5].

Prevalence of these viruses is well known in different populations while data on virus incidence are scarce from countries like Argentina. Only 13 % of studies on influenza-associated hospitalizations, for example, have been published from low- and middle-income countries [2]. Disease burden is part of the information necessary to help ministries of health and the World Health Organization explore the potential value of ARI prevention and control [6]. Furthermore, baseline disease burden data are relevant when evaluating the introduction or expansion of vaccines, infection control measures, and hand washing and respiratory hygiene campaigns which aim to prevent and control respiratory virus transmission [7]. In addition, virus specific incidence can be useful when exploring the potential utility of new vaccines under development that aim to protect children against RSV and other viruses.

Currently, the only respiratory viral vaccines licensed for use among children are influenza vaccines. Safe and effective influenza vaccines are available for preventing influenza illness among children aged >6 months [8]. Although children aged <6 months are ineligible for influenza vaccination, they might be protected if their mothers are vaccinated during pregnancy [9]. The majority of countries in the Americas vaccinate children to prevent influenza illness and its associated economic burden [10, 11]. Historically, countries in the Americas could obtain influenza vaccines through the Pan American Health Organization Revolving Fund for approximately US$4/dose. This has to be administered annually and in 2 doses among children who have not previously been vaccinated. Therefore, although each influenza vaccine dose can be administered for a modest cost, influenza vaccination programs can represent an important annual expenditure for middle-income countries in the Americas.

Argentina, a middle-income country in South America, invests in annual respiratory hygiene campaigns and vaccinates children aged 6 months–2 years to protect them against influenza virus illness [10]. However, information is limited about the value of these investments. Indeed, data regarding the age-stratified rates of respiratory virus illnesses among very young children or their associated costs are unavailable. In this paper, we report on the etiology-specific acute respiratory illness incidence, among a cohort of children aged ≤5 years and costs incurred during their hospitalizations.

## Methods

### Design and study objectives

From June 2008 to December 2010, we conducted a prospective cohort study of acute respiratory infections among children aged ≤5 years living in Buenos Aires, Argentina. A primary objective was to quantify the incidence of laboratory-confirmed influenza among hospitalized children in order to explore the potential value of expanding influenza vaccine use among children in middle-income countries like Argentina. To provide further context to our reader, we also aimed to estimate the incidence of other respiratory viruses among hospitalized children, the cost of hospitalization for acute respiratory illness, the incidence of respiratory viruses among children seeking care in emergency rooms, the prevalence of respiratory virus detections among asymptomatic children, and the impact of applying different commonly used case-definitions to the incidence of respiratory viruses.

### Study population

We followed persons affiliated to Centro de Educación Médica e Investigaciones Clínicas (CEMIC), a non-profit health insurance plan with approximately 1,800 members who are children aged ≤5 years. These children were from primarily middle-income families that paid a monthly fee to CEMIC for preventive health care and for access to its two hospitals and emergency room clinics.

### Enrolment of study children

Medical staff enrolled all CEMIC children who were hospitalized at CEMIC for the management of an acute respiratory infection. Specifically they sought to enroll hospitalized children with any acute respiratory infection sign or symptom (e.g. rhinorrhea, otodynia, cough, wheezing, tachypnea, retractions, or cyanosis). For this study, children with immunosuppression, cardiopathy, and chronic pulmonary, metabolic, and genetic diseases were excluded. The primary goal was to quantify the burden of respiratory viruses among previously healthy community children who did not have severe pre-existing conditions.

Medical staff also enrolled a convenience sample (i.e., an easily accessible, non-random sample) of children presenting to the Saavedra emergency department in Ave. Galván 4102 with any acute respiratory sign or symptom because there were insufficient resources for a random selection. Similarly, medical staff enrolled a convenience sample of asymptomatic children who visited CEMIC for preventive care and denied any respiratory symptom within two weeks prior to their visit. These asymptomatic children could belong to CEMIC or any other health care plan. We did not follow up with the asymptomatic controls after we sampled to determine if they subsequently developed symptoms.

### Demographic and clinical data collection

Parents or guardians were asked by the pediatrician about their children’s demographic and clinical characteristics. Data were recorded in a standardized questionnaire. Data included children’s age, gender, preexisting medical conditions (i.e., prematurity, low birth weight [<2,500 g] [12], reactive airway disease, or atopy), history of breastfeeding, and second-hand exposure to tobacco smoke. In addition, children’s vaccination cards were used to record the status of influenza vaccination and other mandatory vaccines recommended by Argentina Ministry of Health (e.g. haemophilus influenza type B, hepatitis B and oral polio vaccine) [13]. Medical staff recorded children’s respiratory signs and symptoms at the time of presentation. For hospitalized patients, data about clinical course of hospitalization, including length of stay and oxygen therapy, admission to an intensive care unit, and mechanical ventilation were recorded.

### Sample collection and respiratory virus detection

Respiratory samples (i.e., nasopharyngeal aspirates from hospitalized children and nasopharyngeal swabs from outpatients) were obtained from children who presented within 5 days of symptom onset. Samples were placed in viral transport media at ~4 °C and were immediately sent to CEMIC Virology Laboratory to be processed for rapid antigen detection by immunofluorescence (IF) [1]. RSV, adenovirus (AdV), influenza A (FluA), influenza B (FluB), and parainfluenza (PIV) 1, 2, or 3 were detected by indirect IF with monoclonal antibodies (Millipore Corporation, Massachusetts USA); human metapneumovirus was detected by direct IF (bioMérieux, France). An immunofluorescence assay was unavailable for rhinovirus. Therefore, an aliquot of the original sample was stored at −70 °C for rhinovirus detection by real-time RT-PCR. Viral RNA/DNA extraction was manually performed by using the QIAamp®MinElute® Virus Spin (Qiagen GmbH, Germany), according to the manufacturer’s recommendations. Real-time RT-PCR was performed by amplifying a segment of the 5’ non-coding region of the genome, in a LightCycler® 2.0 (Roche Diagnostics, France) [14].

### Incidence calculations

To estimate the annual incidence of acute respiratory infections, we divided the number of CEMIC children admitted at CEMIC hospital with an acute respiratory infection by the number of children who were members of the CEMIC plan in 2008, 2009, and 2010 [15]. We assumed each child enrolled in the CEMIC plan was at risk of developing an acute respiratory infection and subsequent complications requiring hospitalization. CEMIC children would predominantly seek care at the CEMIC hospital because their families prepaid for such care through the plan. We assumed each child who was a member of the CEMIC plan during 2008 contributed ½ year of person-time to the analysis because the study started in June-December of 2008. Each child who was a member of the CEMIC plan during 2009 and 2010 contributed two years of person-time to the analyses because medical staff searched for these admissions year-round. Finally, we determined the proportion of children admitted to the hospital with any acute respiratory sign or symptom that met commonly used respiratory case-definitions (Table 1) to determine their impact on incidence calculations.

**Table 1:** Operationalization of commonly used case-definitions

• Severe acute lower respiratory infection: hospitalized children who had cough or difficulty breathing (i.e., having tachypnea, or oxygen requirement) with retractions [3]
• Severe acute respiratory infection: hospitalized children with subjective fever and cough [27]
• Influenza-like illness: children who had sought care in emergency room with subjective fever and cough [22]

We also used a multiplier to estimate the incidence of emergency room visits for acute respiratory infections model [6] because we did not have the resources to enroll all children seeking care at the emergency room. First we reviewed emergency room records to determine the number of CEMIC children who sought care for acute respiratory illnesses each month during 2008–2010. We assumed that CEMIC children seeking care for acute respiratory signs and symptoms had approximately the same probability of testing positive for a specific respiratory virus than all children sampled by convenience during the same month and year [16]. We estimated the number of CEMIC children that would have tested positive for respiratory viruses if all had enrolled in the study by multiplying the monthly number of CEMIC children seeking care at emergency room by the proportion that provided respiratory samples and subsequently tested positive for viruses from CEMIC and other health plans. We divided this numerator by the person-time CEMIC children were enrolled during the June 2008-December 2010 study period. Finally, we stratified our incidence estimates by age-group (<6 months, 6–23 months, and 2–5 years) because we anticipated the proportion of CEMIC children positive for respiratory viruses and the subsequent rates might differ statistically by these age strata [17].

### Costs associated with hospitalization

Administrative hospital records were examined for all hospitalized children with acute respiratory illnesses to determine room, laboratory, consumables, and physician costs incurred by the pooled CEMIC account [18]. These costs represented the amount of money CEMIC spent to provide in-hospital care within the context of its non-for-profit status. We converted CEMIC cost data in Argentinean pesos to U.S. dollars using the average Argentina’s Central Bank rate during June 2008–December 2010.

## Ethics

The cohort study protocol was reviewed and approved by the CEMIC Institutional Review Board (No. 00001745 IORG001315). Parents or guardians signed informed consent forms to allow their children to participate in this investigation. The Centers for Disease Control and Prevention (CDC) reviewed the protocol for human subjects’ protection and determined to be non-research because CDC staff was involved only after information had been collected and only analyzed non- identifiable data.

## Results

There were 1,729 children aged ≤ 5 years enrolled in CEMIC in 2008, 1,838 in 2009 and 1,893 in 2010 (4,739 child-years of follow-up). During this study period, we identified 89 hospitalizations (18.8/1000 child-years) and 8,925 emergency room visits (1.9/child-years) among CEMIC children aged ≤ 5 years. We obtained detailed clinical information from all 89 hospitalized children and 269 (3 %) of the 8,925 children who visited the emergency room (89+ 269 = 358 study children) (Table 2). Hospitalized children were admitted for an average of median of 3 days; IQR, 2–4 days). One third (90) of children seeking care at emergency room belonged to the CEMIC plan and 179 belonged to other insurance plans. In addition, medical staff obtained nasopharyngeal swabs from 37 asymptomatic control children.

**Table 2:** Demographic and clinical characteristics of hospitalized, emergency room, and asymptomatic control children in Buenos Aires during 2008–2010

	Hospitalized	Emergency room		Controls
	*N* = 89	CEMIC *n* = 90	Other *n* = 179	*n* = 37
Age (months)	10^a^	13	19	43^b^
Males^c^	57	56	56	70
Preexisting conditions				
Reactive airway disease^c^	39	37	43	24
Tobacco exposure^c^	21	19	16	14
Late preterm birth^c^	19	13	13	16
Atopic dermatitis^c^	13	23	23	16
Birth weight <2,500 g	11	6	8	11
Vaccines and breastfeeding				
Influenza vaccine if age >6 months^c^	31	37	50	26
Up-to-date with other vaccines^c^	97	100	97	97
Breastfed (months)	5	6	6	6
Sign and Symptom				
Fever >38°C^c^	65	83	73	
Cough^c^	92	89	87	
Tachypnea^c^	84^a^	45	42	
Retractions^c^	76^a^	24	20	
Wheezing^c^	70^a^	41	34	
Vomiting^c^	15	19	16	
Diarrhea^c^	10	10	7	
Cyanosis^c^	2	1	1	
Diagnoses				
Rhinitis^c^	81^a^	89	91	
Bronchiolitis^c^	74^a^	38	31	
Pneumonia^c^	14^a^	5	7	
Otitis^c^	8	9	7	
Pharyngitis^c^	6^a^	36	35	
Bronchitis^c^	1	10	7	
Conjunctivitis^c^	0^a^	23	13	

^a^Statistical difference between hospitalized versus emergency room children in rank-sum or *Χ* ^2^ test, *p* < 0.05

^b^Statistical difference between ill (hospitalized and emergency room) children and control children in Wilcoxon rank-sum test, *p* < 0.001

^c^Percentage

Hospitalized children were younger (median age, 10 months; IQR, 4–23 months) than emergency room children (median age, 18 months; IQR, 8–33 months) and asymptomatic controls (median age, 43 months; IQR, 24–60 months). Hospitalized children were also more likely to present with tachypnea, retractions, and wheezing and be diagnosed with bronchiolitis or pneumonia than emergency room children (Table 2) (*Χ*^2^*p* < 0.03). Conversely, children were less likely to be diagnosed with rhinitis, pharyngitis, or conjunctivitis during their hospitalization than emergency room children (*Χ*^2^*p* < 0.04). There were no significant differences between CEMIC and non-CEMIC children seeking care at emergency room (Table 2).

### Laboratory findings

**Fig. 1:**
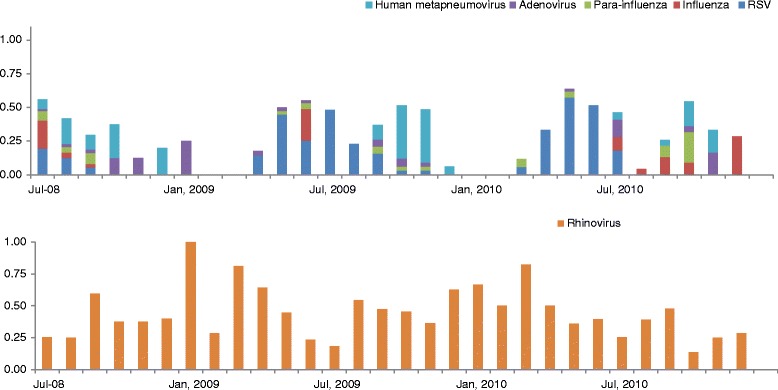
Proportion of children testing positive for respiratory viruses each month during 2008–2010 From July 2008 to December 2010, respiratory samples from Argentina children aged ≤5 years, who were hospitalized or who sought care at an emergency room with any acute respiratory infection sign or symptom (e.g., rhinorrhea, cough, wheezing, tachypnea, retractions, or cyanosis), were taken and tested for several respiratory viruses (Human metapneumovirus, adenvirus, Para-influenza, influenza, RSV and rhinovirus). While rhinoviruses were detected throughout the year, RSV, human metapneumovirus, influenza, adenovirus, and parainfluenza 1 through 3 viruses were also identified starting April or May, with peak activity during June or July, during Argentina’s austral winter

**Fig. 2:**
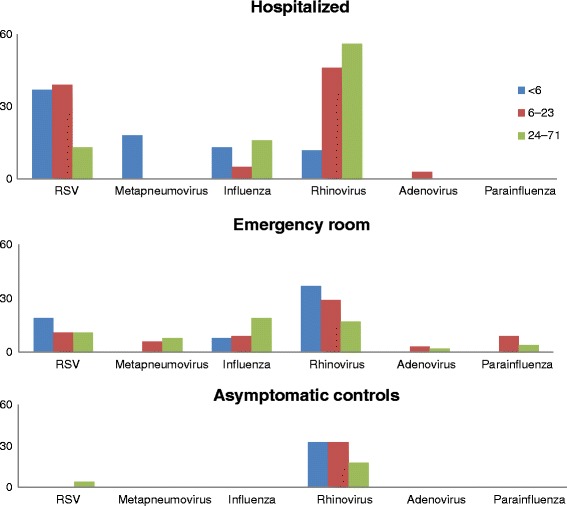
Proportion of hospitalized, emergency room, and asymptomatic control children testing positive for respiratory viruses, 2008–2010. From July 2008 to December 2010, respiratory samples from Argentina children aged ≤5 years, who were hospitalized or who sought care at an emergency room with any acute respiratory infection sign or symptom (e.g., rhinorrhea, cough, wheezing, tachypnea, retractions, or cyanosis), were taken and tested for several respiratory viruses (Human metapneumovirus, adenvirus, Para-influenza, influenza, RSV and rhinovirus). RSV, human metapneumovirus, and influenza were frequently identified among hospitalized children aged <6 months (37, 18, 13 %, respectively) but seldom among asymptomatic controls (<3 %). Rhinovirus, however, was identified among 40 % of hospitalized children and 22 % of asymptomatic controls

All 358 hospitalized and emergency room children and 37 asymptomatic controls provided a nasopharyngeal aspirate or swab. While rhinoviruses were detected throughout the year, RSV, human metapneumovirus, influenza, adenovirus, and parainfluenza 1 through 3 viruses were also identified starting April or May, with peak activity during June or July, during Argentina’s austral winter (Fig. 1). RSV, human metapneumovirus, and influenza were frequently identified among hospitalized children aged <6 months (37, 18, 13 %, respectively) but seldom among asymptomatic controls (<3 %) (Fig. 2). Rhinovirus, however, was identified among 40 % of hospitalized children and 22 % of asymptomatic controls (Table 3). Only five patients tested positive for two viruses: two children were positive for RSV and influenza, one for rhinovirus and RSV, one for rhinovirus and parainfluenza, and one for rhinovirus and adenovirus.

**Table 3:** Number^a^ (and percentage) of hospitalized, emergency room, and asymptomatic control children testing positive for respiratory viruses by age group in months during 2008–2010

	Hospitalized				Emergency room				Asymptomatic control children			
	<6	6–23	24–71	0–71	<6	6–23	24–71	0–71	<6	6–23	24–71	0–71
RSV	9 (37)	14 (39)	3 (13)	26 (31)	7 (19)	16 (11)	12 (11)	35 (12)	0 (0)	0 (0)	1 (4)	1 (3)
Metapneumovirus	3 (18)	0 (0)	0 (0)	3 (5)	0 (0)	8 (6)	8 (8)	16 (6)	0 (0)	0 (0)	0 (0)	0 (0)
Influenza	3 (13)	2 (5)	4 (16)	9 (10)	3 (8)	13 (9)	21 (19)	37 (13)	0 (0)	0 (0)	0 (0)	0 (0)
Rhinovirus	2 (12)	13 (46)	10 (56)	25 (40)	13 (37)	40 (29)	18 (17)	71 (26)	1 (33)	2 (33)	5 (18)	8 (22)
Adenovirus	0 (0)	1 (3)	0 (0)	1 (1)	0 (0)	4 (3)	2 (2)	6 (2)	0 (0)	0 (0)	0 (0)	0 (0)
Parainfluenza	0 (0)	0 (0)	0 (0)	0 (0)	0 (0)	13 (9)	4 (4)	17 (6)	0 (0)	0 (0)	0 (0)	0 (0)
Any virus_b_	17 (71)	27 (67)	17 (68)	61 (69)	21 (58)	89 (61)	60 (55)	170 (58)	1 (33)	2 (33)	6 (21)	9 (24)

From July 2008 to December 2010, respiratory samples from Argentina children aged ≤5 years, who were hospitalized or who sought care at an emergency room with any acute respiratory infection sign or symptom (e.g., rhinorrhea, cough, wheezing, tachypnea, retractions, or cyanosis), were taken and tested for several respiratory viruses (Human metapneumovirus, adenovirus, Para-influenza, influenza, RSV and rhinovirus). RSV, human metapneumovirus, and influenza were frequently identified among hospitalized children aged <6 months (37, 18, 13 %, respectively) but seldom among asymptomatic controls (<3 %). Rhinovirus, however, was identified among 40 % of hospitalized children and 22 % of asymptomatic controls. Co-infections were an uncommon event in our study. Only five patients tested positive for two viruses: two children were positive for RSV and influenza, one for rhinovirus and RSV, one for rhinovirus and parainfluenza, and one for rhinovirus and adenovirus (results not shown)

^a^Although all children for tested for all viruses, some samples yielded unreadable results. The unreadable results were excluded from the analysis. Therefore, there was some variation in the denominator for each virus

^b^The number of viruses detected in each column may not add up to the number of children with at least one virus because some children had coinfections

Children with laboratory-confirmed RSV typically (>70 %) had presumptive diagnoses of bronchiolitis while 56 % of those with laboratory-confirmed influenza, had presumptive diagnoses of influenza illness. There were no statistical differences in the proportion of CEMIC children testing positive for specific respiratory viruses each month at emergency room when compared to the proportion of emergency room children from other health plans (*Χ*^2^*p* = 0.8). All 63 (71 %) of 89 hospitalized CEMIC children for who their clinicians ordered blood cultures as part of routine clinical care, tested negative for bacterial pathogens.

### Incidence

The incidence of RSV (24/1000 children-years), influenza (8/1000 children-years) and human metapneumovirus (8/1000 children-years) illnesses was highest among hospitalized children aged <6 months and decreased among older children (Table 4). In contrast, the incidence of rhinovirus was highest (12/1000 children-years) among those aged 6–23 months. The incidence of these respiratory viruses seemed lower when we limited the analyses to children who met the severe acute lower respiratory infection or severe acute respiratory infection case-definitions. For example, while the incidence of RSV respiratory illnesses was 6/1000 children-years among hospitalized children aged ≤5 years, only half of these met the severe acute lower respiratory infection case-definition (3/1000 children-years). In emergency rooms, the incidence of rhinovirus was 459 (95 % CI, 121–869)/1,000 children-years; RSV 352 (95 % CI, 167–557); influenza 185 (95 % CI, 57–333); parainfluenza 177 (95 % CI, 15–400); metapneumovirus 130 (95 % CI, 7–276); and adenovirus 73 (95 % CI, 2–180). Eighty-three percent of children seeking care at emergency room for laboratory-confirmed influenza illness met the ILI case definition.

**Table 4:** Incidence of respiratory viruses per 1000 hospitalized children per year, 2008–2010

	Hospitalized with at least one respiratory sign or symptom				Severe acute lower respiratory infection				Severe acute respiratory infection			
	<6	6–23	24–71	All	<6	6–23	24–71	All	<6	6–23	24–71	All
RSV	24	13	1	6	13	11	0.4	4	13	10	0.7	4
Metapneumovirus	8	-	-	1	5	-	-	0.5	3	-	-	0.2
Influenza	8	2	1	2	3	-	0.4	0.5	3	1	1	1
Adenovirus	-	1	-	0.2	-	0.9	-	0.2	-	1	-	0.2
Parainfluenza	-	-	-	-	-	-	-	-	-	-	-	-
Rhinovirus	5	12	4	6	3	8	3	4	3	5	2	3

The incidence of RSV (24/1000 children-years), influenza (8/1000 children-years) and human metapneumovirus (8/1000 children-years) illnesses was highest among hospitalized children aged <6 months and decreased among older children. In contrast, the incidence of rhinovirus was highest (12/1000 children-years) among those aged 6–23 months

### Hospitalization costs

The total cost of hospitalization to CEMIC was a median of US$529 (IQR, US$362–789). Fifty-nine percent of the total cost was comprised room charges (median US$310 [IQR US$227–517]). Consumables represented 13 % (median US$ 66 [IQR US$38–129); diagnostic assays, 12 % (median US$64 [IQR US$54–99]); medications, 10 % (median US$54 [IQR US$27–148]); and consults with specialists, 6 % (median US$30 [IQR US$0–60]) of the total costs.

## Discussion

Our findings suggest that respiratory viruses including rhinovirus, RSV, influenza, and human metapneumovirus in particular may have caused a substantial disease and economic burden among Argentinean children aged ≤5 years. This RSV burden was highest among the youngest children with an incidence similar to that documented in other countries [3]. Indeed, very young children are more likely to require hospitalization as a result of viral respiratory illness [19]. Although RSV vaccines are under development, none are licensed for use among children. The national program in Argentina recommends that palivizumab prophylaxis be given to all preterm children with a gestational age of < 29 weeks or to preterm children with a gestational age of 29–32 weeks and bronchopulmonary dysplasia during RSV epidemics to decrease the probability of severe respiratory infections. To improve the value of palivizumab, surveillance staff should provide pediatricians with timely information about RSV epidemics in their community, especially during the austral winter when such epidemics are anticipated.

In our study, approximately ten percent of hospitalized and emergency room children had influenza infections. The incidence of influenza-related hospitalizations was substantial (2/1,000 children-years) and comparable with that of sites that estimated the seasonal and 2009 pandemic influenza incidence, and invest in influenza vaccine programs [20]. The cost associated with hospitalization was also substantial (~US$500/hospitalization) and would represent a little less than 1/2 of the median monthly net household income in Argentina during 2008–2010 (US$1235/month [IQR US$1102–1366) [21]. Indeed, the cost of hospitalization would have been even higher among children admitted to intensive care where the length of stay was an average of 8 days rather than 3 days like in the general pediatric ward.

In our study, approximately 30 % of children aged >6 months had been vaccinated against influenza. Southern hemisphere vaccines were well-matched to the predominant influenza strains characterized in Argentina during the study period, except during the 2009 influenza A (H1N1) pandemic [22]. It is reasonable to assume that the influenza burden among CEMIC children would have been lower if influenza vaccine coverage had been higher [23]. Institutions like CEMIC should explore how to improve influenza vaccine utilization among children aged > 6 months such that it is administered on par with that of other pediatric vaccines. Argentina adopted pneumococcal vaccination in 2012 [24] and institutions like CEMIC may also want to explore the additional impact of pneumococcal and influenza vaccination on early childhood morbidity.

Rhinoviruses were frequently detected in our pediatric population. Rhinoviruses are among the most frequent causes of the common cold and have been associated with lower respiratory infections [25]. Previously, we reported that rhinovirus had a prevalence of 27 % among outpatient children [14] and 47 % among hospitalized children aged ≤5 years in Argentina [1]. In our current study, we detected rhinovirus among 2/5 of hospitalized children and 1/5 of asymptomatic controls. In our study, a substantive number of children had a severe respiratory illnesses requiring hospitalization that seem to have been precipitated by rhinovirus infections. We were unable to determine which proportion of these hospitalizations was directly attributable to rhinovirus illness because we did not follow asymptomatic children prospectively to determine if they subsequently developed illness and because these children were typically older than hospitalized children. Indeed, given the frequency of rhinovirus infections and our lack of follow up among control children, it was not possible to determine whether rhinovirus positive asymptomatic children were shedding virus from an illness that occurred >2 weeks before their enrolment, were currently asymptomatic but about to develop signs and symptoms of clinical illness, or had a subclinical infection. More data are needed to fully understand the natural history of children with rhinovirus infections, the clinical impact of rhinoviruses and the potential value of antivirals and vaccines targeting these viruses [26].

During our statistical analyses, we noted that our incidence estimates varied substantially depending on the case-definitions we used. For example, while the incidence of laboratory-confirmed influenza among children hospitalized for the management of an illness with at least one sign or symptom of respiratory infection was 2/1000 children-years, the incidence was 1/1000 children-years among those who met the severe acute lower respiratory infection or severe acute respiratory infection case-definitions [27]. Similarly 4/5 of children at emergency room with laboratory-confirmed influenza illness met the ILI case-definition typically used by influenza surveillance platform throughout the Americas [22]. Investigators seeking to estimate the burden of RSV, influenza, human metapneumovirus, and other respiratory viruses should be aware of the impact case-definitions have on their estimates depending on the age of group studied and local health seeking practices [6].

Our cohort study had several important limitations. We enrolled children without underlying conditions because these represented the majority of the CEMIC population. Aside from acute respiratory infections, participating children were otherwise healthy. It is likely that our incidence estimates would have been higher if we had also enrolled children with comorbidities such as pulmonary dysplasia, immunodeficiencies and cardiopathies because these are known risk factors for complications and hospitalizations from respiratory virus illnesses [8]. RSV, human metapneumovirus, influenza, parainfluenza 1–3, and adenovirus were detected through direct antigen detection by immunofluorescence. This low-cost assay is readily available throughout Latin America [27] but has a lower sensitivity than polymerase chain reaction (PCR) [28]. It is therefore possible that the incidence of these viruses may have been higher if we had tested all samples through PCR. We were also unable to randomly sample asymptomatic control children from both study sites of the same age and during the same epidemiologic week as hospitalized case-patients because of limited resources and because CEMIC parents seldom provided consent for nasopharyngeal swabs if their children were asymptomatic during preventive care visits. As a result, asymptomatic control children were typically older than cases and consequently had a lower probability of testing positive for key respiratory viruses (e.g., RSV). Last, we obtained 2 ½ years of respiratory specimens from a convenience sample of 1 % of emergency room patients, a subpopulation that may not have been representative of all CEMIC children, the 8,925 children who sought emergency room care during the study period, or children throughout Argentina during 2008–2010.

## Conclusions

Our study suggests that the incidence of rhinovirus, RSV, human metapneumovirus and influenza among hospitalized and emergency room children was substantive. Children aged <6 months were most commonly affected during Argentina’s austral winter. Respiratory virus illnesses were associated with costly hospitalizations. Institutions like CEMIC should explore the potential value of promoting hand washing, cough etiquette, and other non-pharmaceutical interventions aimed at interrupting respiratory virus transmission among families with very young children. Pending the availability of vaccines for RSV and other respiratory viruses, CEMIC and the Argentina Ministry of Health could also explore the impact of influenza vaccination among pregnant women and children aged 6 months to 5 years as recommended by the World Health Organization’s Strategic Advisory Group of Experts on Immunization [29].

## Abbreviations

AdV: Adenovirus
ARI: Acute respiratory infection
CEMIC: Centro de Educación Médica e Investigaciones Clínicas
CDC: U.S. Centers for Disease Control and Prevention
CI: Confidence interval
FluA: Influenza A
FluB: Influenza B
HMPV: Human metapneumovirus
If: Immunofluorescence
IQR: Interquartile range
PIV: Parainfluenza virus
RSV: Respiratory syncytial virus
RT-PCR: Reverse transcription polymerase chain reaction
US/USA: United States of America
